# National Veterinary College

**Published:** 1896-06

**Authors:** 


### NATIONAL VETERINARY COLLEGE.

THE fourth annual commencement exercises took place at the College Building, April 11th, at 8 P.M. Speeches were made by Profs. Salmon, Willets, and Lockwood. The first college prize was awarded to E. E. Seitz, of Pennsylvania. Honorable mention was made of J. W. Petty, of North Carolina; W. R. Jobson, of Pennsylvania; and B. E. Harper, of Washington, D. C. Reid R. Ashworth won the Junior prize for scholarship. The following gentlemen received the college degree, that of D.V.S.: P. A. Fisk, D.Sc., of New York; E. E. Graffam, of Washington, D. C.; R. H. Hadfield, of Washington, D. C.; B. E. Harper, of Washington, D. C.; J. M. Heagerty, of Maryland; W. R. Jobson, of Pennsylvania; C. H. Lockwood, of Washington, D. C.; J. W. Petty, of North Carolina; C. H. Morrison, of Connecticut; F. H. Schneider, of Pennsylvania; E. E. Seitz, of Pennsylvania; John Lockwood, D.V.S., of Washington, D. C.

